# Expression of mucoid induction factor MucE is dependent upon the alternate sigma factor AlgU in *Pseudomonas aeruginosa*

**DOI:** 10.1186/1471-2180-13-232

**Published:** 2013-10-18

**Authors:** Yeshi Yin, F Heath Damron, T Ryan Withers, Christopher L Pritchett, Xin Wang, Michael J Schurr, Hongwei D Yu

**Affiliations:** 1 Department of Biochemistry and Microbiology, Joan C. Edwards School of Medicine at Marshall University, Huntington, WV 25755, USA; 2 Department of Pediatrics, Joan C. Edwards School of Medicine at Marshall University, Huntington, WV 25755, USA; 3 Institute of Plant Protection and Microbiology, Zhejiang Academy of Agricultural Sciences, No. 198, Shiqiao Road, Hangzhou 310021, China; 4 Progenesis Technologies, LLC, 1111 Veterans Memorial Blvd, Huntington, WV 25701, USA; 5 Department of Microbiology, School of Medicine, University of Colorado, Aurora, Colorado 80045, USA; 6 Department of Health Sciences, East Tennessee State University, Johnson City, TN 37615, USA; 7 Division of Infectious Diseases and International Health, University of Virginia, Box 800419, MR-6, Charlottesville, VA 22908, USA

**Keywords:** *Pseudomonas aeruginosa*, Alginate, Mucoidy, AlgU/T, MucE, Sigma factor

## Abstract

**Background:**

Alginate overproduction in *P. aeruginosa*, also referred to as mucoidy, is a poor prognostic marker for patients with cystic fibrosis (CF). We previously reported the construction of a unique mucoid strain which overexpresses a small envelope protein MucE leading to activation of the protease AlgW. AlgW then degrades the anti-sigma factor MucA thus releasing the alternative sigma factor AlgU/T (σ^22^) to initiate transcription of the alginate biosynthetic operon.

**Results:**

In the current study, we mapped the *mucE* transcriptional start site, and determined that P_*mucE*_ activity was dependent on AlgU. Additionally, the presence of triclosan and sodium dodecyl sulfate was shown to cause an increase in P_*mucE*_ activity. It was observed that *mucE*-mediated mucoidy in CF isolates was dependent on both the size of MucA and the genotype of *algU*. We also performed shotgun proteomic analysis with cell lysates from the strains PAO1, VE2 (PAO1 with constitutive expression of *mucE*) and VE2Δ*algU* (VE2 with in-frame deletion of *algU*). As a result, we identified nine *algU*-dependent and two *algU*-independent proteins that were affected by overexpression of MucE.

**Conclusions:**

Our data indicates there is a positive feedback regulation between MucE and AlgU. Furthermore, it seems likely that MucE may be part of the signal transduction system that senses certain types of cell wall stress to *P. aeruginosa*.

## Background

*P. aeruginosa*, a Gram-negative bacterium, is the leading cause of morbidity and mortality in patients with cystic fibrosis (CF) [1]. In CF, *P. aeruginosa* is often isolated from sputum samples and exhibits a phenotype called mucoidy, which is due to overproduction of an exopolysaccharide called alginate. It is also an environmental bacterium which normally does not overproduce alginate [2]. The emergence of mucoid *P. aeruginosa* isolates in CF sputum specimens signifies the onset of chronic respiratory infections. Mucoidy plays an important role in the pathogenesis of *P. aeruginosa* infections in CF, which includes, but is not limited to: increased resistance to antibiotics [1], increased resistance to phagocytic killing [3,4] and assistance in evading the host’s immune response [3].

A major pathway for the conversion to mucoidy in *P. aeruginosa* is dependent upon AlgU (AlgT, σ^22^), an alternative sigma factor that drives transcription of *algD* encoding the key enzyme for alginate biosynthesis [5,6]. Previous studies have shown that several genes take part in the regulation of AlgU activation and alginate overproduction. MucA is a trans-membrane protein that negatively regulates mucoidy by acting as an anti-sigma factor via sequestering AlgU to the cytoplasmic membrane [7]; MucB and intra-membrane proteases AlgW, MucP and ClpXP were reported to affect alginate production by affecting the stability of MucA [8]. A small envelope protein called MucE was found to be a positive regulator for mucoid conversion in *P. aeruginosa* strains with a wild type MucA [9]. The mechanism for *mucE* induced mucoidy is due to its C-terminal –WVF signal, which can activate the protease AlgW possibly by interaction with the PDZ domain [9]. Upon activation, AlgW initiates the proteolytic degradation of the periplasmic portion of MucA, causing the release of AlgU to drive expression of the alginate biosynthetic operon [9]. While the function of MucE as an alginate inducer was identified, its physiological role, and its role in the regulation of mucoidy in clinical isolates, remains unknown.

Comparative analysis through Basic Local Alignment Search Tool (BLAST) using the genomes of *Pseudomonas* species from the public databases reveals that MucE orthologues are found only in the strains of *P. aeruginosa*[9]. In order to study the role and regulation of MucE in *P. aeruginosa*, we first mapped the *mucE* transcriptional start site. We then examined the effect of five different sigma factors on the expression of *mucE in vivo*. Different cell wall stress agents were tested for the induction of *mucE* transcription. Expression of MucE was also analyzed in non-mucoid CF isolates to determine its ability to induce alginate overproduction.

## Methods

### Bacteria strains, plasmids, and growth conditions

Bacterial strains and plasmids used in this study are shown in Additional file 1: Table S1. *E. coli* strains were grown at 37°C in Luria broth (LB, Tryptone 10 g/L, Yeast extract 5 g/L and sodium chloride 5 g/L) or LB agar. *P. aeruginosa* strains were grown at 37°C in LB or on *Pseudomonas* isolation agar (PIA) plates (Difco). When required, carbenicillin, tetracycline or gentamicin were added to the growth media. The concentration of carbenicillin, tetracycline or gentamycin was added at the following concentrations: for LB broth or plates 100 μg ml^-1^, 20 μg ml^-1^ or 15 μg ml^-1^, respectively. The concentration of carbenicillin, tetracycline or gentamycin to the PIA plates was 300 μg ml^-1^, 200 μg ml^-1^ or 200 μg ml^-1^, respectively.

### The *mucE* primer extension assay

Total RNA was isolated from *P. aeruginosa* PAO1 grown to an OD_600_ of 0.6 in 100 ml LB at 37°C as previously described [10]. The total RNA was isolated using the RNeasy kit (Qiagen, Valencia, CA) per the manufacturer’s instructions. Primers for *mucE* (PA4033), seq 1 (5′-CCA TGG CTA CGA CTC CTT GAT AG-3′) and seq 2 (5′-CAA GGG CTG GTC GCG ACC AG-3′), were radio-labeled using T4 polynucleotide kinase (New England Biolabs, Ipswich, MA) and γP^32^-ATP. Primer extensions were performed using the Thermoscript RT-PCR system (Invitrogen, Carlsbad, CA) with either PA4033 seq 1 or seq 2 with 10–20 μg of total RNA. Extensions were performed at 55°C for an hour. Primer extension products then were electrophoresed through a 6% acrylamide/8M urea gel along with sequencing reactions (Sequenase 2.0 kit, USB, Cleveland, OH) using the same primers used in the extension reactions.

### Transformation and conjugation

*E. coli* One Shot TOP10 cells (Invitrogen) were transformed via standard heat shock method according to the supplier’s instructions. Plasmid transfer from *E. coli* to *Pseudomonas* was performed via triparental conjugations using the helper plasmid pRK2013 [11].

### Generating PAO1 miniCTX-P_*mucE*_-*lacZ* reporter strain

PAO1 genomic DNA was used as a template to amply 618 bp upstream of the start site (ATG) of *mucE* using two primers with built-in restriction sites, HindIII-*mucE*-P-F (5′-AAA GCT TGG TCG TTG AAA GTC TGC ACC TCA-3′) and EcoRI-*mucE*-P-R: (5′-CGA ATT CGG TTG ATG TCA CGC AAA CGT TGG C-3′). The P_*mucE*_ amplicon was TOPO cloned and digested with HindIII and EcoRI restriction enzymes before ligating into the promoterless *Pseudomonas* integration vector miniCTX-*lacZ*. The promoter fusion construct miniCTX-P_*mucE*_-*lacZ* was integrated onto the *P. aeruginosa* chromosome of strain PAO1 at the CTX phage *att* site [12] following triparental conjugation with *E. coli* containing the pRK2013 helper plasmid [11].

### Screening for a panel of chemical agents that can promote P_*mucE*_ transcription

Membrane disrupters and antibiotics were first tested by serial dilution to determine the minimum inhibitory concentration (MIC) for strain PAO1::*attB*::P_*mucE*_-*lacZ*. An arbitrary sub-MIC concentration for each compound was then tested for the induction effect through the color change of 5-Bromo-4-chloro-3-indolyl β-D-galactopyranoside (X-gal, diluted in dimethylformamide to a concentration of 4% (w/v)). The final concentration of the compounds used in this study are listed as follows: triclosan 25 μg/ml, tween-20 0.20% (v/v), hydrogen peroxide 0.15%, sodium hypochlorite 0.03%, SDS 0.10%, ceftazidimine 2.5 μg/ml, tobramycin 2.5 μg/ml, gentamicin 2.5 μg/ml, colisitin 2.5 μg/ml, and amikacin 2.5 μg/ml. PAO1::*attB*::P_*mucE*_-*lacZ* was cultured overnight in 2 ml LB broth, 10 μl of overnight culture and 10 μl of 4% X-gal was added to each treatment culture tube (2 ml LB broth + cell wall stress agent). The cultures were grown overnight at 37°C with shaking at 150 rpm and were used to visually observe the change of the color. LB broth lacking X-gal was used as a negative control.

### The β-galactosidase activity assay

*Pseudomonas* strains were cultured at 37°C on three PIA plates. After 24 hours, bacterial cells were harvested and re-suspended in PBS. The OD_600_ was measured and adjusted to approximately 0.3. Cells were then permeabilized using toluene, and β-galactosidase activity was measured at OD_420_ and OD_550_. The results in Miller Units were calculated according to this formula: Miller Units = 1000 × [OD_420_ - (1.75 × OD_550_)]/[Reaction time (minutes) × Volume (ml) × OD_600_] [13]. The reported values represent an average of three independent experiments with standard error.

### Alginate assay

*P. aeruginosa* strains were grown at 37°C on PIA plates in triplicate for 24 hrs or 48 hrs. The bacteria were collected and re-suspended in PBS. The OD_600_ was analyzed for the amount of uronic acid in comparison with a standard curve made with D-mannuronic acid lactone (Sigma-Aldrich), as previously described [14].

### iTRAQ^®^ MALDI TOF/TOF proteome analysis

Strains PAO1, VE2 and VE2Δ*algU* were cultured on PIA plates for 24 hrs at 37°C. Protein preparation and iTRAQ mass spectrometry analysis was performed according to previously described methods [15].

## Results

### Mapping of the *mucE* promoter in PAO1

**Figure 1:**
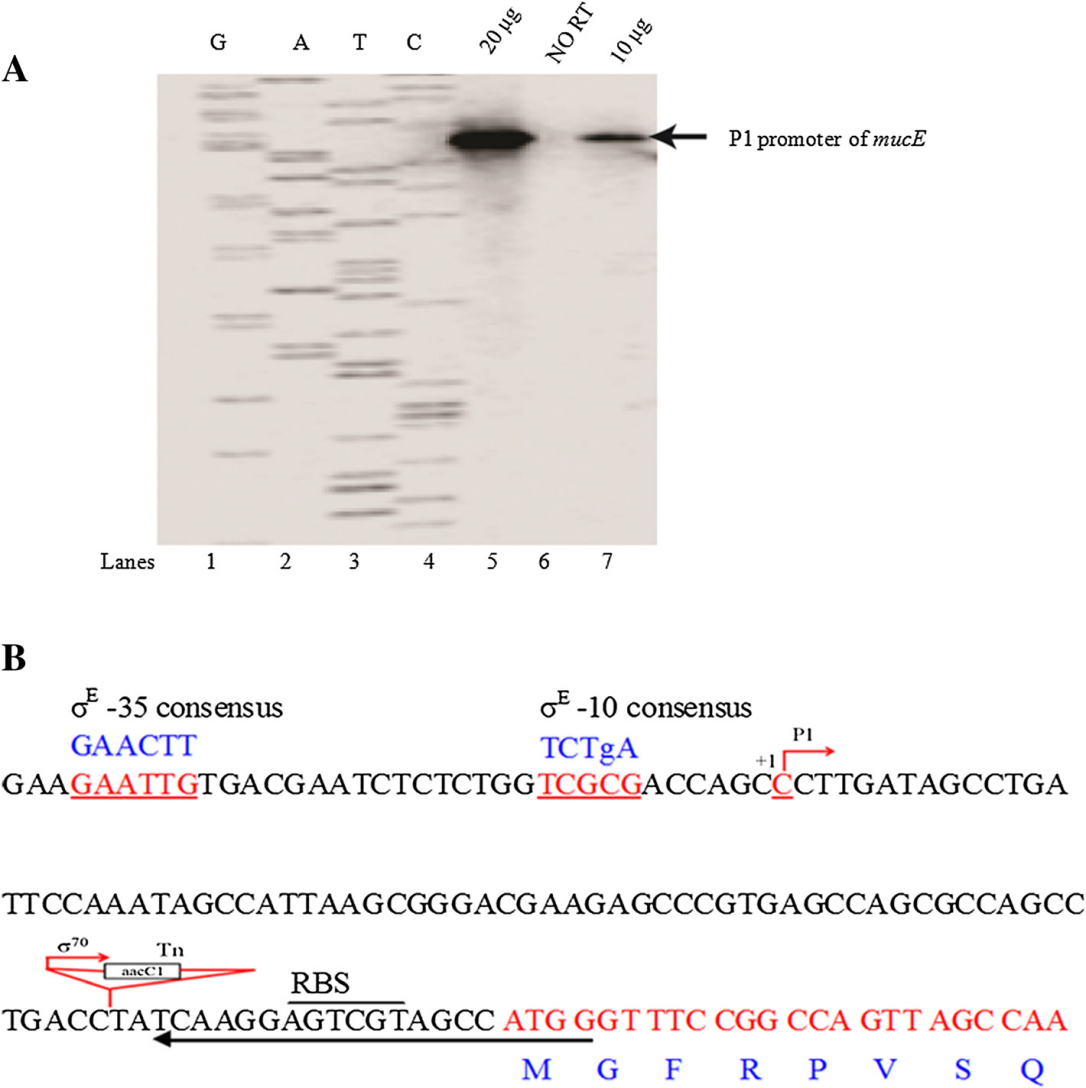
**Mapping of the *mucE *transcriptional start site in *P. aeruginosa *PAO1. A)** Primer extension mapping of mRNA 5′ end. Total RNA was isolated from the non-mucoid PAO1. The conditions used for labelling of primers for *mucE* are described in Methods. The primer extension product was run adjacent to the sequencing ladder generated with the same primer as highlighted in the *mucE* sequence. The arrow indicates the position of the P1 transcriptional start site of *mucE*. **B)** The *mucE* promoter sequence in strains PAO1 and PAO1VE2. The transposon (Tn) insertion site of PAO1VE2 is underlined along with the putative ribosome binding site (RBS) for *mucE*. In strain PAO1VE2, the gentamicin resistance cassette (*aacC1*) gene carries a σ^70^ dependent promoter. The arrow pointing leftward corresponds to the position of primer seq 1 used for mapping the P1 start site.

We previously identified MucE, a small envelope protein, which induces mucoid conversion in *P. aeruginosa* when overexpressed [9]. Induction of MucE activates the intramembrane protease AlgW resulting in activation of the cytoplasmic sigma factor AlgU and conversion from nonmucoidy to mucoidy in strains with a wild type MucA [9]. Stable production of copious amounts of alginate is characteristic of strain VE2 which carries a mariner transposon insertion before *mucE*[9]. This insertion is likely responsible for the constitutive expression of the *mucE* gene [9]. However, it is unclear how *mucE* is naturally expressed in parent PAO1. To determine this, primer extension analysis of the *mucE* promoter region was performed. With higher amounts of PAO1 RNA (20 μg), we observed one prominent transcriptional start site that is initiated 88 nucleotides upstream of the *mucE* translational start site (Figure 1). This suggests that, under these conditions, *mucE* has one promoter that is active in PAO1.

### The alternative sigma factor AlgU activates transcription of *mucE in vivo*

**Figure 2:**
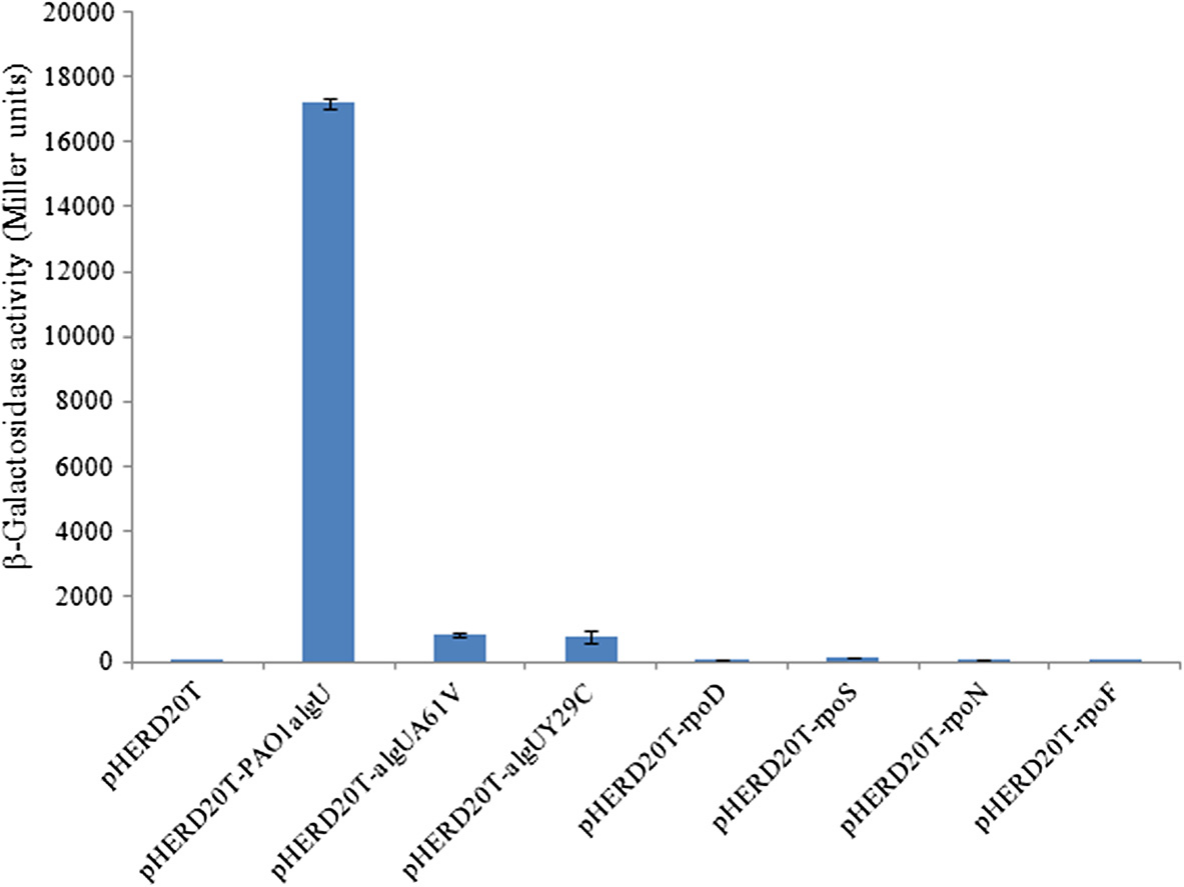
**Effect of overexpression of sigma factors on the P**_***mucE***_**expression.** The sigma factors AlgU, RpoD, RpoN, RpoS and RpoF were expressed from an arabinose-inducible promoter in pHERD20T [16], and the P_*mucE*_ activity was determined via β-galactosidase assay from a merodiploid strain of PAO1 carrying P_mucE_-*lacZ* integrated on the chromosome. The values reported in this figure represent an average of three independent experiments with standard error.

**Figure 3:**
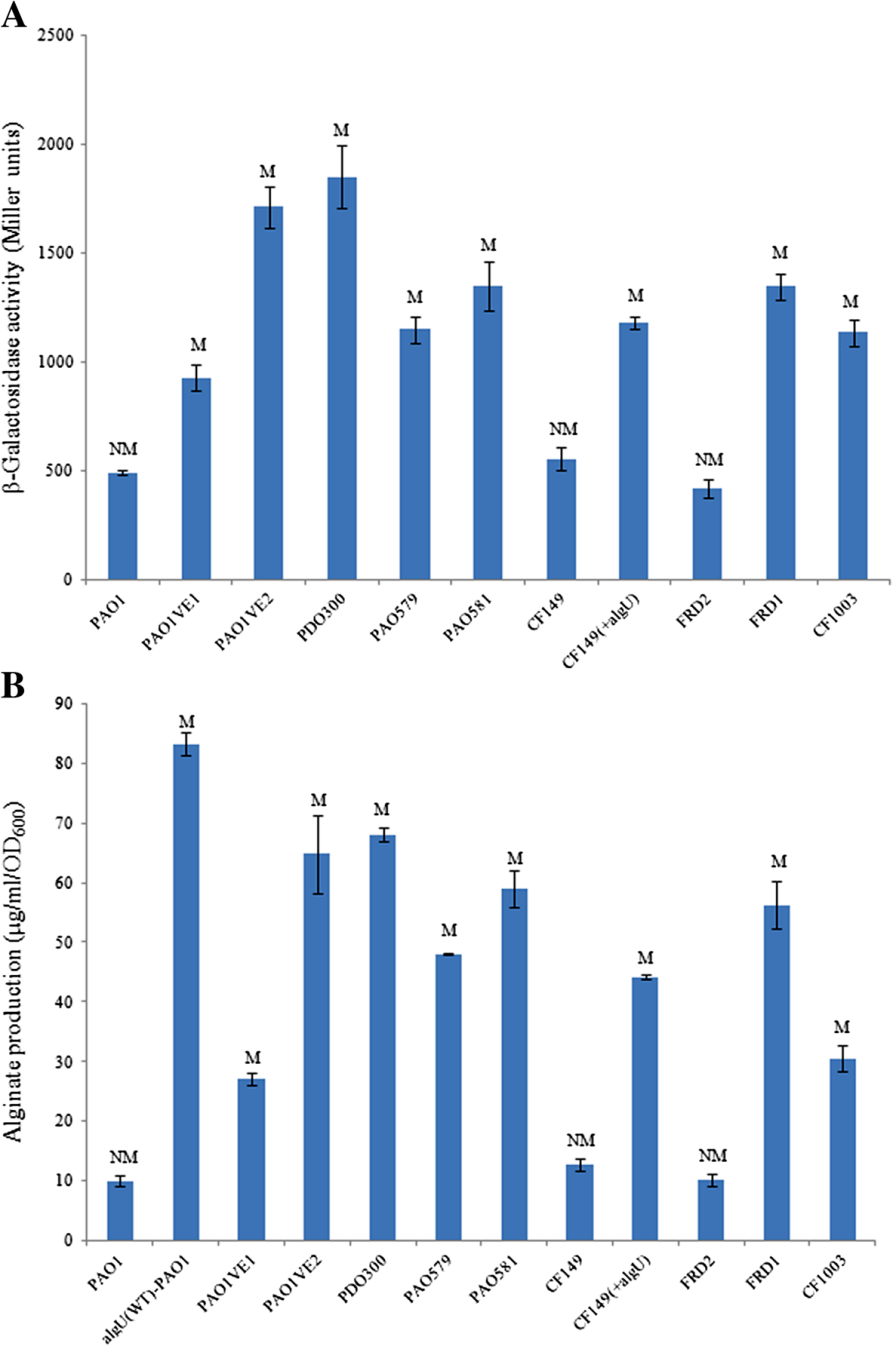
**Correlation between the P**_***mucE***_**activity and alginate overproduction in various strains of *P. aeruginosa*. A)** Measurement of the P_*mucE*_ activity in various mucoid laboratory and clinical strains. **B)** Measurement of alginate production (μg/ml/OD_600_) by the same set of strains as in A grown on PlA plates without carbenicillin for 24 h at 37°C. The *algU*(WT)-PAO1 represents the PAO1 strain contained the pHERD20T-*algU*(WT). The values reported in this figure represent an average of three independent experiments with standard error.

In order to determine which sigma factor is responsible for driving *mucE* transcription, miniCTX-P_*mucE*_-*lacZ* was integrated onto the PAO1 chromosome. To identify the sigma factor that activates the expression of P_*mucE*_, we expressed *P. aeruginosa* sigma factors (RpoD, RpoN, RpoS, RpoF and AlgU) *in trans* and measured P_*mucE*_*-lacZ* activity in this PAO1 fusion strain. As seen in Figure 2, Miller assay results showed that AlgU significantly increased the promoter activity of P_*mucE*_ in PAO1. However, we did not observe any significant increases in promoter activity of P_*mucE*_ with other sigma factors tested in this study. As stated earlier, AlgU is a sigma factor that controls the promoter of the alginate biosynthetic gene *algD*[5,6]. In order to determine whether the activity of P_*mucE*_ is elevated in mucoid strains, pLP170-P_*mucE*_ was conjugated into mucoid laboratory and clinical *P. aeruginosa* strains. As seen in Figures 3A and 3B, the activity of P_*mucE*_ increased in mucoid laboratory and CF isolates.

### Cell wall stress promotes expression of *mucE* from P_*mucE*_

**Figure 4:**
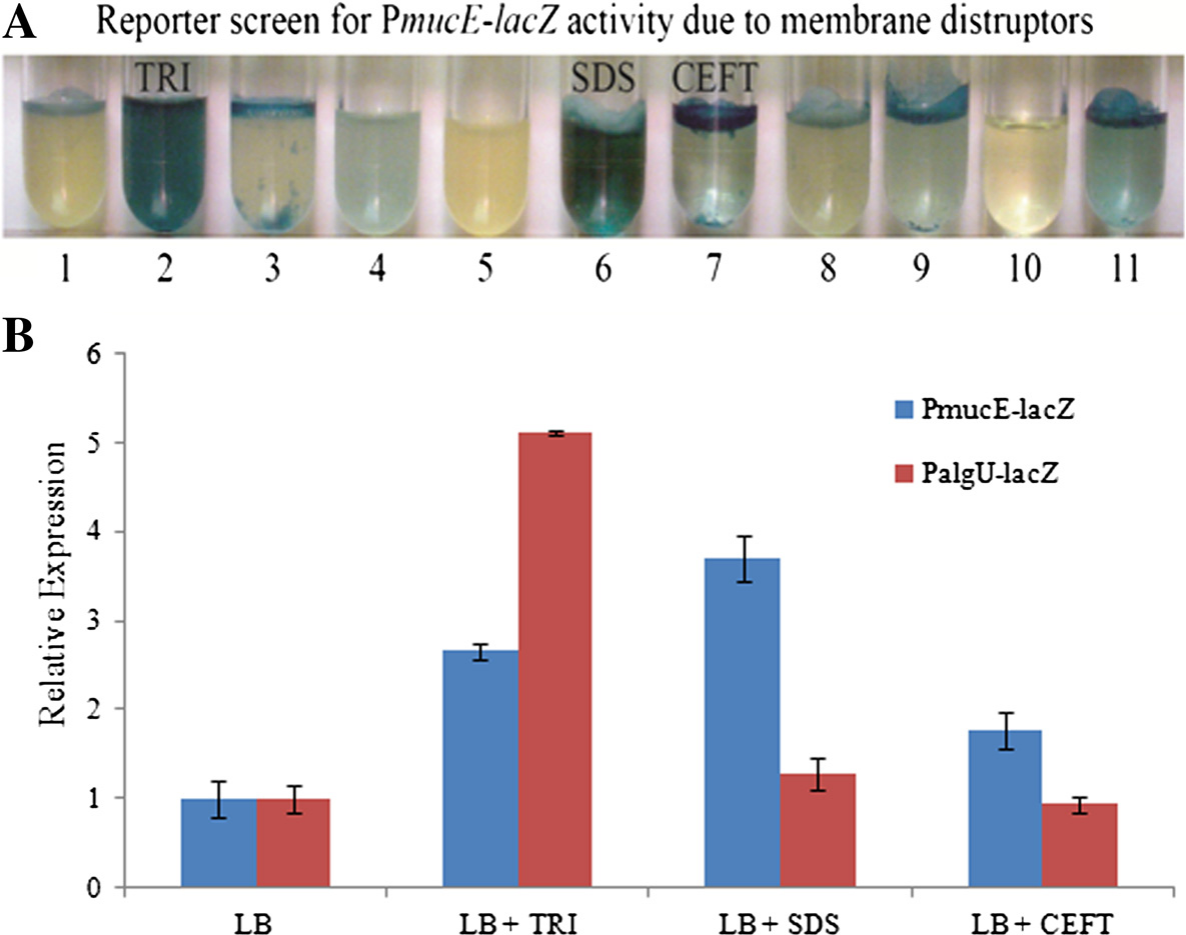
**Induction of P**_***mucE***_**activity by cell wall stress. A**. A 1/200 dilution of the PAO1::*attB*::P_*mucE*_-*lacZ* recombinant strain grown overnight was inoculated into LB media containing X-gal and the agents listed as follows, 1) LB (control), 2) triclosan 25 μg/ml, 3) tween-20 0.20% (v/v), 4) hydrogen peroxide 0.15%, 5) bleach 0.03%, 6) SDS 0.10%, 7) ceftazidimine 2.5 μg/ml, 8) tobramycin 2.5 μg/ml, 9) gentamicin 2.5 μg/ml, 10) colisitin 2.5 μg/ml, and 11) amikacin 2.5 μg/ml. **B**. Triclosan, SDS, and ceftazidimine were tested for the induction of the P_*mucE*_ and P_*algU*_ promoters. The activities of the promoter fusions were measured by β-galactosidase activity as described in Methods.

Since the *mucE* promoter was active in nonmucoid PAO1 and further increased in mucoid cells (Figure 3A), the conditions that induce *mucE* expression were examined. To do this, we used the same P_*mucE*_-*lacZ* strain of PAO1 to measure the activation of *mucE* by some compounds previously shown to cause cell wall perturbations [17,18]. The phenotypes of strains harboring the P_*mucE*_-*lacZ* fusion in the presence of various cell wall stress agents are shown in Figure 4A. While sodium hypochlorite and colistin didn’t induce a visual change in P_*mucE*_ activity, three compounds, triclosan, sodium dodecyl sulfate (SDS) and ceftazidime induced marked expression of P_*mucE*_-*lacZ* in PAO1. Each resulted in elevated levels of β-galactosidase activity as indicated by the blue color of the growth media. This suggests that the P_*mucE*_ promoter activity was increased in response to these stimuli (Figure 4A). Miller assays were performed to measure the changes in P_*mucE*_-*lacZ* activity due to these compounds. Triclosan increased P_*mucE*_-*lacZ* activity by almost 3-fold over LB alone (Figure 4B). An increase in P_*mucE*_-*lacZ* should increase P_*algU*_-*lacZ* activity. As expected, triclosan caused a 5-fold increase in P_*algU*_-*lacZ* activity. However, SDS and ceftazidime increased the P_*mucE*_-*lacZ* activity, but did not promote the P_*algU*_-*lacZ* activity (Figure 4B).

### Alginate production is reduced in the *mucE* mutant compared to PAO1

Expression of *mucE* can cause alginate overproduction [9]. However, we wondered if *mucE* would affect transcriptional activity at P_*algU*_ and P_*algD*_ promoters. In order to determine this, both pLP170-P_*algU*_ and pLP170-P_*algD*_ with each promoter fused to a promoterless *lacZ* gene were conjugated into PAO1 and PAO1VE2, respectively. As seen in Additional file 1: Figure S1, the activity of P_*algU*_ (PAO1VE2 vs. PAO1: 183,612.04 ± 715.23 vs. 56.34 ± 9.68 Miller units) and P_*algD*_ (PAO1VE2 vs PAO1: 760,637.8 ± 16.87 vs. 138.18 ± 9.68 Miller units) was significantly increased in the *mucE* over-expressed strain PAO1VE2. Although, Qiu *et al*. [9] have reported that AlgU is required for MucE induced mucoidy, we wanted to know whether MucE is required for AlgU induced mucoidy. As seen in Additional file 1: Figure S2, we did not observe that the over-expression of MucE induced mucoidy in PAO1Δ*algU*. This result is consistent with what was previously reported by Qiu *et al.*[9]*.* However, the alginate production induced by AlgU was decreased in the *mucE* knockout strain. The alginate production induced by AlgU in two isogenic strains, PAO1 and PAO1*mucE*::ISphoA/hah is 224.00 ± 7.35 and 132.81 ± 2.66 μg/ml/OD_600_, respectively (Additional file 1: Figure S2). These results indicate that alginate overproduction in PAO1 does not require MucE. However, MucE can promote the activity of AlgU resulting in a higher level of alginate production in PAO1 compared to the *mucE* knockout. Previously, Boucher *et al.*[19] and Suh *et al.*[20] have reported that sigma factors RpoN and RpoS were involved in alginate regulation. In order to determine whether *mucE* induced mucoidy was also dependent on other sigma factors besides AlgU, pHERD20T-*mucE* was conjugated and over-expressed in PAO1Δ*rpoN*, PAO1*rpoS*::ISlacZ/hah and PAO1*rpoF*::ISphoA/hah. The results showed that the *mucE* induction caused mucoid conversion in PAO1*rpoS*::ISlacZ/hah and PAO1*rpoF*::ISphoA/hah when 0.1% L-arabinose was added to the media. However, 0.5% L-arabinose was required for mucoid conversion in PAO1Δ*rpoN*. The alginate production induced by MucE in PAO1*rpoS*::ISlacZ/hah, PAO1*rpoF*::ISphoA/hah and PAO1Δ*rpoN* is 150.62 ± 5.27, 85.53 ± 4.10 and 31.84 ± 0.25 μg/ml/OD_600,_ respectively. These results suggested that RpoN, RpoS and RpoF are not required for MucE-induced mucoidy in PAO1. Conversely, over-expression of these sigma factors *rpoD*, *rpoN*, *rpoS* and *rpoF* did not induce mucoid conversion in PAO1. When the strains of PAO1 with sigma factor overexpression were measured for alginate production, the level is as follows: 5.11 ± 1.25 (+*rpoD*), 13.07 ± 4.16 (+*rpoN*), 3.50 ± 0.10 (+*rpoS*) and 7.68 ± 1.23 (+*rpoF*) μg/ml/OD_600_.

### MucE-induced mucoidy in clinical CF isolates is based on two factors, size of MucA and genotype of *algU*

**Figure 5:**
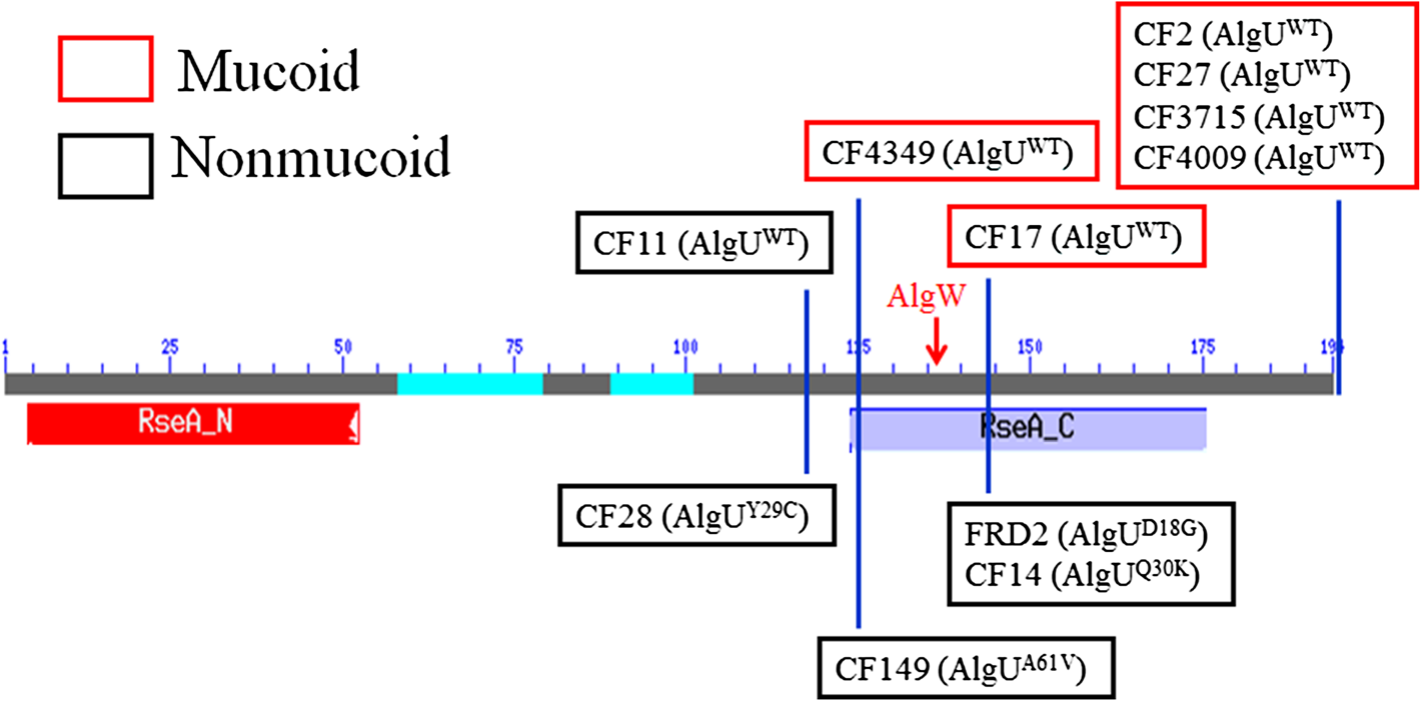
**MucE-mediated mucoid conversion in nonmucoid clinical isolates is dependent on MucA length and *algU *genotype.** The length of MucA is shown with two functional domains as depicted with RseA_N and RseA_C, which represent the N-terminal domain of MucA predicted to interact with AlgU in the cytoplasm and C-terminal domain of MucA located in the periplasm, respectively. The domain prediction is based on the NCBI Conserved Domain Database (CDD). The blue vertical line represents the truncated MucA due to the mutation from each CF strain relative to the full length of wild type MucA. The type of AlgU is indicated for each CF strain (WT or mutant with the indicated change of amino acid due to missense mutation). Those strains that become mucoid upon *mucE* induction are shown in red, while those that remain nonmucoid are shown in black. The red arrow indicates the cutting site of MucA by AlgW. pHERD20T-*mucE* was conjugated into these non-mucoid CF isolates, and then incubated on PIA plates containing carbenicillin and 0.1% L-arabinose at 37°C for 24 hours. Mucoid or non-mucoid phenotype was scored based on visual inspection and the amount of alginate production. The quantity of alginate was measured and shown in Table S2.

Although, Qiu *et al*. [9] have reported that over-expression of *mucE* can induce mucoidy in laboratory strains PAO1 and PA14, its ability to induce mucoidy in clinical CF isolates has not been investigated. Particularly, *mucE*’s relationship to *mucA* mutations is unknown since different mutations would result in production of MucA with various molecular masses. To test if the length of MucA had an effect on MucE-mediated mucoid induction, we selected a group of nonmucoid clinical isolates and observed any phenotypic change after overexpression of *mucE*. Figure 5 summarizes the results. First, strains with wild type AlgU and MucA became mucoid. Although, MucA of CF2 carries a missense mutation, CF2 became mucoid. Secondly, as seen in Figure 5 and Additional file 1: Table S2, *mucE* could induce mucoidy in CF17 (MucA^143 + 3 aa^) and CF4349 (MucA^125 + 3 aa^) with wild type AlgU, but not in strains containing *algU* carrying a missense mutation [CF14 (MucA^143 + 3 aa^), FRD2 (MucA^143 + 3 aa^) and CF149 (MucA^125 + 3 aa^)]. Thirdly, overexpression of *mucE* did not induce mucoidy in CF11 and CF28, whose MucA length was 117aa, despite a wild type AlgU in CF11. These results suggest that MucE-mediated mucoidy is dependent on the combination of two factors, MucA length and *algU* genotype (Figure 5). The effect of MucE on mucoid induction is more obvious in strains with MucA length up to 125 amino acid residues coupled with wild type AlgU, but missense mutations in AlgU can significantly reduce the potency of MucE.

### Mutant AlgUs display partial activity resulting in decreased amount of alginate

**Figure 6:**
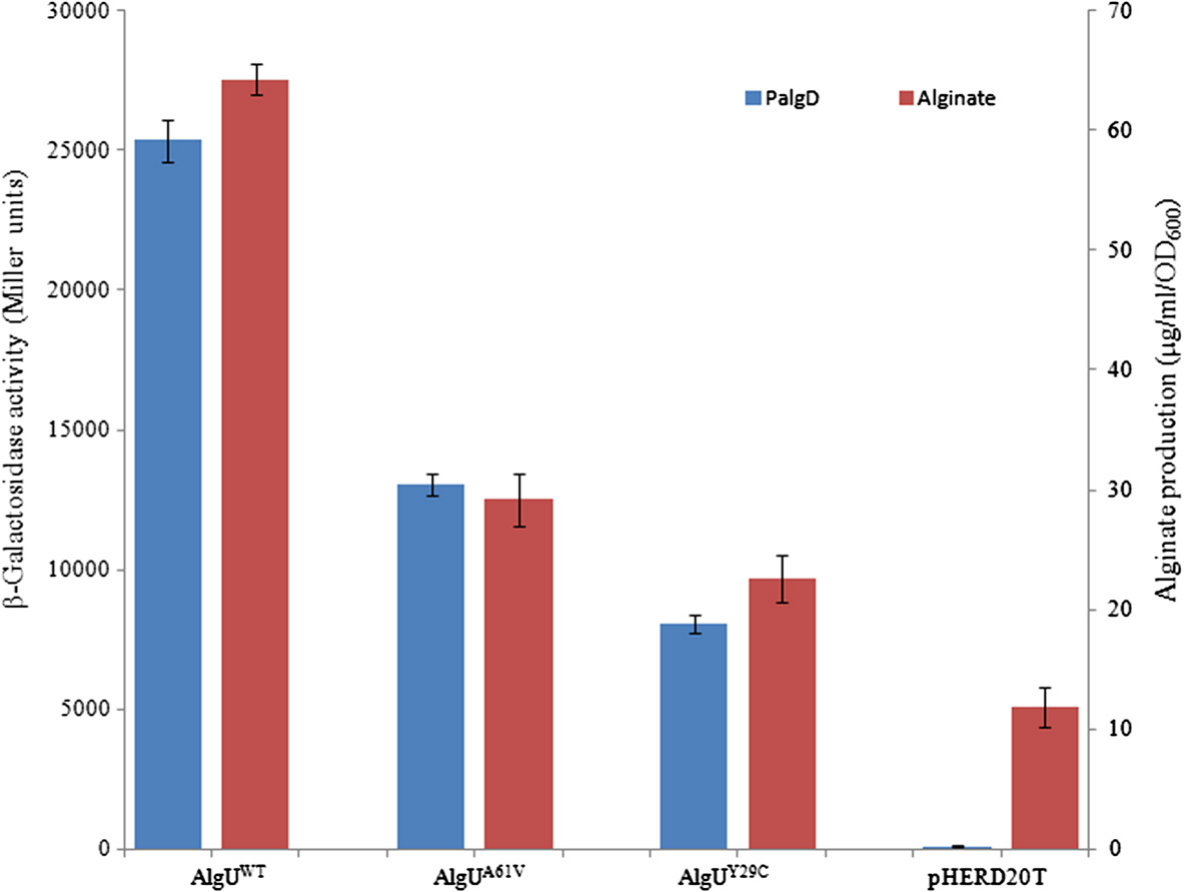
**AlgU with missense mutations induces decreased amount of alginate compared to wild type AlgU.** PAO1, CF149 and CF28 *algUs* were cloned into pHERD20T vector, and conjugated into PAO1Δ*algU* and PAO1miniCTX-P_*algD*_-*lacZ*, respectively. Alginate production (μg/ml/OD_600_) and P_*algD*_ activity were measured after culture overnight on PIA plates supplemented with 300 μg/ml of carbenicillin. The values reported here represent an average of three independent experiments with standard error.

Schurr *et al*. have reported that second-site suppressor mutations in *algU* can affect mucoidy [21]. DeVries and Ohman [22] also reported that mucoid-to-nonmucoid conversion in alginate-producing *P. aeruginosa* is often due to spontaneous mutations in *algT* (*algU*). Recently, Damkiaer *et al*. [23] showed that point mutations can result in a partially active AlgU. To test whether the activity of AlgU from different CF isolates is affected due to mutation, the CF149 and CF28 *algU* genes were cloned and over-expressed in PAO1Δ*algU* and PAO1miniCTX-P_*algD*_-*lacZ*, respectively. As seen in Figure 6, these constructs retained the ability to promote the transcription of P_*algD*_ and alginate production. Also, when transposon libraries were screened for mucoid revertants in CF149 [24] and FRD2, three and five mucoid mutants in CF149 and FRD2, respectively, were identified due to transposon insertion before *algU* causing the overexpression of *algU* (data not shown). However, the activity of the mutant AlgU is lower than that of wild type AlgU (Figure 6). In order to determine whether the mutant AlgU still has the ability to promote *mucE* transcription, *algU* genes from CF149 and CF28 were cloned into pHERD20T, respectively, and over-expressed in PAO1 miniCTX-P_*mucE*_-*lacZ* strain. As seen in Figure 2, mutant forms of AlgU were still able to promote *mucE* transcription, albeit at a reduced level.

### Characterization of the MucE regulon using iTRAQ analysis

In order to determine the effect of *mucE* expression on the proteome change, we performed iTRAQ proteome analysis via MALDI TOF/TOF. Total protein lysates of PAO1, VE2 (PAO1 with constitutive expression of *mucE*) and VE2Δ*algU* (VE2 with in-frame deletion of *algU*) were collected and analyzed. Within the three samples, 166 unique proteins were identified with 1455 peptides assayed at/or above 95% confidence. The data set was then filtered to include only proteins that were significantly different between samples and the number of the detected peptides for each protein more than three (Additional file 1: Table S3). By comparing the proteomes of VE2 to PAO1, the effects of increased MucE levels on PAO1 were examined; while comparing VE2Δ*algU* to PAO1 allowed for the determination of AlgU-independent protein production in VE2. As seen in Additional file 1: Table S3, compared to PAO1, 11 proteins were differentially expressed due to *mucE* over-expression, and two of them (elongation factor Tu and transcriptional regulator MvaT) are AlgU-independent.

## Discussion

**Figure 7:**
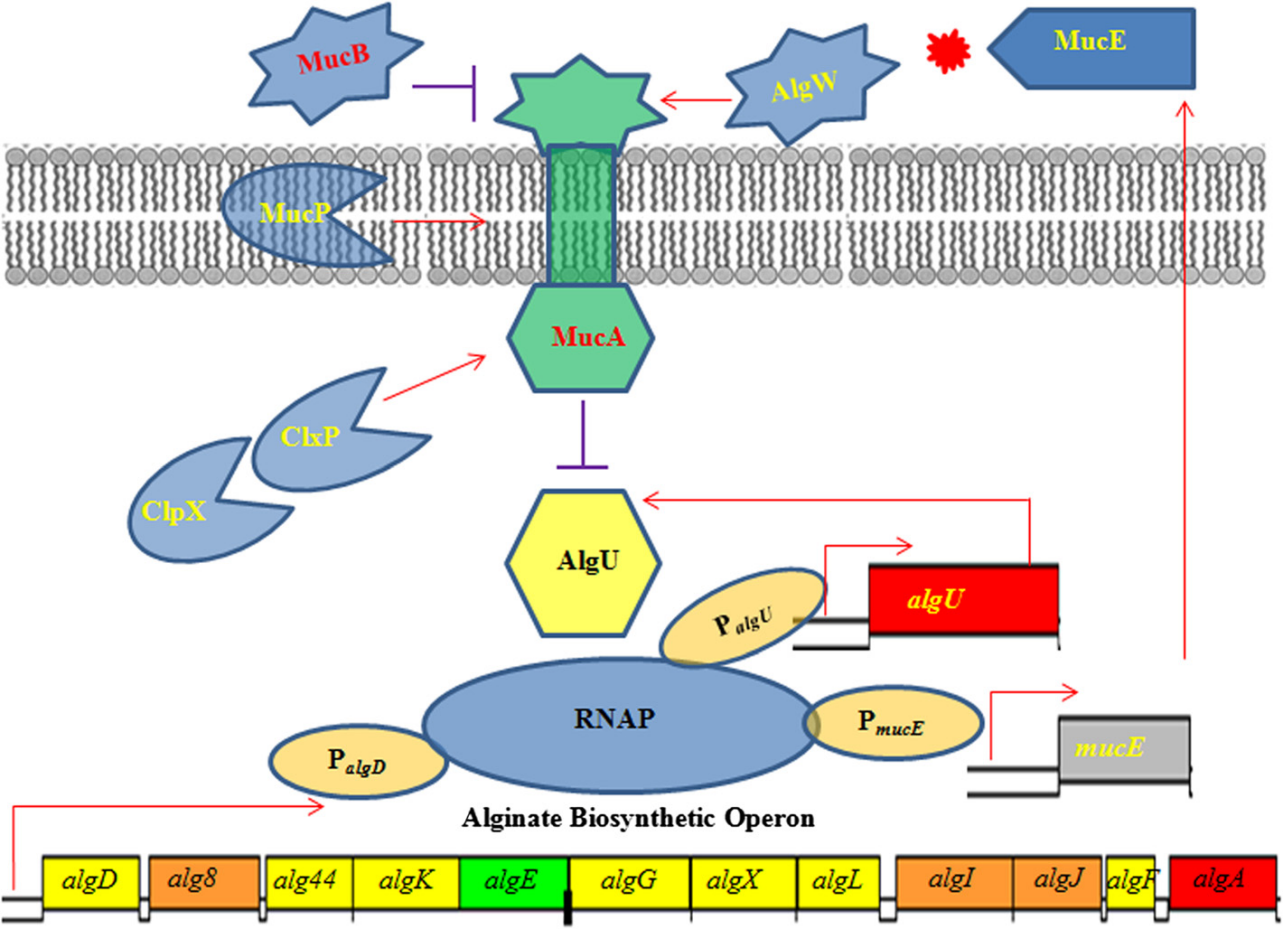
**Schematic diagram summarizing the positive feedback between MucE and AlgU and their relationship to alginate overproduction.** AlgU is an alternative sigma factor that controls the alginate biosynthetic operon. Additionally, AlgU regulates itself, as well as drives transcription of *mucE*. MucE has the C-terminal –WVF motif that can activate the protease AlgW, thereby causing the degradation of the anti-sigma factor MucA. The degradation of MucA results in the release of AlgU to activate transcription at the P_*algU*,_ P_*algD*_ and P_*mucE*_ promoter sites.

MucE is a small envelope protein whose overexpression can promote alginate overproduction in *P. aeruginosa* strains with a wild type MucA [9]. Here, we observed that AlgU can induce the expression from P_*mucE*_, and consistent with this result, the P_*mucE*_ activity is higher in mucoid strains than in non-mucoid strains (Figure 3). AlgU is a stress-related alternate sigma factor that is auto-regulated from its multiple promoters [25]. As a sigma factor, AlgU drives transcription of the alginate biosynthetic gene *algD*[5] and the alginate regulator gene *algR*[26]. As shown in this study, AlgU can also activate the transcription of *mucE*, and subsequently, depending on the level of induction, MucE can increase P_*algU*_ and P_*algD*_ activity resulting in mucoid conversion in clinical strains. Together, these results suggest a positive feedback mechanism of action in which AlgU activates *mucE* expression at the P_*mucE*_ promoter, and in return, the increased level of MucE can increase AlgU activity by activating AlgW, which further degrades MucA (Figure 7). This regulation between MucE and AlgU probably ensures that a cell, upon exposure to stress, can rapidly reach the desired level of AlgU and alginate production. Therefore, it is not surprising to see that a higher level of alginate production requires *mucE* in *P. aeruginosa* strains with a wild type MucA (Additional file 1: Figure S2). We also noted that some cell wall stress agents, like triclosan and SDS can induce the expression of *mucE*. However, the differential activation at P_*algU*_ by triclosan but not SDS suggests SDS may not be an inducer at P_*algU*_, and/or the stimulation by SDS was not high enough to initiate the positive feedback regulation of MucE by AlgU. Nevertheless, this observation is consistent with what was previously reported by Wood *et al.* regarding the absence of induction at P_*algD*_ by SDS [27]. Furthermore, we found that strain PAO1 does not become mucoid when cultured on LB or PIA plates supplemented with triclosan or SDS at the concentration as used in Figure 4 (data not shown).

Qiu *et al.* have reported that MucE can induce alginate overproduction when over-expressed *in vivo*[9]. However, nothing was known about the regulation of *mucE*. Recently, the genome-wide transcriptional start sites of many genes were mapped by RNA-seq in *P. aeruginosa* strain PA14 [28]. However, the transcriptional start site of the *mucE* gene (PA14_11670) was not included. In this study, we reported the mapping of the *mucE* transcriptional start site. Furthermore, we found the transcription of *mucE* is dependent on AlgU. Analysis of the upstream region of *mucE* reveals an AlgU promoter-like sequence (Figure 1). Previously, Firoved *et al.* identified 35 genes in the AlgU regulon, based on scanning for AlgU promoter consensus sequence (GAACTTN_16-17_TCtgA) in the PAO1 genome [26]. In this study, we found that AlgU can activate the transcription of *mucE*. In order to determine whether AlgU can bind to P_*mucE*_ region, AlgU was purified (Additional file 1: Figure S3) and electrophoretic mobility shift assay (EMSA) was performed. As seen in Additional file 1: Figure S4, our results showed that AlgU affected the mobility of P_*mucE*_ DNA, especially in the presence of *E. coli* RNA polymerase core enzyme, suggesting a direct binding of AlgU to P_*mucE*_. However, whether small regulatory RNAs or other unknown regulator proteins are also involved in the transcriptional regulation of *mucE* needs further study. LptF is another example of an AlgU-dependent gene, but doesn’t have the consensus sequence in the promoter region [29]. While MucE, as a small envelope protein is positively regulated through a feedback mechanism, it’s not clear how many AlgU-regulated genes follow the same pattern of regulation as MucE.

The *mucA* mutation is a major mechanism for the conversion to mucoidy. Mutation can occur throughout the *mucA* gene (585 bps) [30]. These mutations result in the generation of MucA proteins of different sizes. For example, unlike the wild type MucA with 194 amino acid residues, MucA25, which is produced due to a frameshift mutation, results in a protein containing the N-terminal 59 amino acids of MucA, fused with a stretch of 35 amino acids without homology to any known protein sequence [31]. MucA25 lacks the trans-membrane domain of wild type MucA, predicting a cytoplasmic localization. Therefore, different *mucA* mutations could possibly result in different cellular compartment localization. Identification of MucE’s function as an inducer of alginate in strains with wild type MucA and AlgU strongly suggests MucE acts through interaction with AlgW in the periplasm. On the other hand, the loss of this predicted MucA-AlgW interaction can be seen in two strains, CF11 and CF28, which lack the major cleavage site of AlgW [32] (Figure 5). Interestingly, we observed that the missense mutation in *algU* can reduce, but not completely abolish, the activity of AlgU as an activator for alginate production. This data may explain why mutant *algU* alleles have reduced P_*mucE*_ activity (Figure 2). Furthermore, since AlgU is an auto-regulated protein [25], this may explain why the P_*mucE*_ activity induced by mutant AlgU is lower than that of wild type AlgU. A slightly higher activity of P_*mucE*_ noted in CF149(+*algU*) than in PAO1VE1 (Figure 3A) could be due to a combined effect of dual mutation of *algU* and *mucA* in CF149. In strains of FRD2 and CF14, the retention of the AlgW cleavage site is not sufficient to restore mucoidy. This is because of the partial function of AlgU, which can be seen with alginate production and AlgU-dependent P_*algD*_ promoter activity (Figure 6). Altogether, these results suggest that mucoidy in clinical isolates can be modulated by a combination of two factors, the size of the MucA protein and the genotype of the *algU* allele in a particular strain. MucA size determines its cellular localization and its ability to sequester AlgU, and the *algU* allele determines whether AlgU is fully or partially active.

The iTRAQ results showed that the expression of two proteins was significantly increased and the expression of nine proteins was decreased in the *mucE* over-expressed strain VE2 (Additional file 1: Table S3). Of these 11 proteins, nine of them are AlgU dependent, for including flagellin type B. Garrett *et al.* previously reported that AlgU can negatively regulate flagellin type B and repress flagella expression [33]. However, no AlgU consensus promoter sequences were found within the upstream of the 11 regulated genes through bioinformatics analysis, indicating that these may be indirect effect. In addition, two proteins (elongation factor Tu and transcriptional regulator MvaT) were significantly decreased when compared to PAO1 proteome, but remained unchanged when comparison was made between VE2 and VE2ΔalgU, suggesting the reduction of these two proteins was independent of AlgU in the MucE over-expressed strain. MvaT is a global regulator of virulence in *P. aeruginosa*[34], and elongation factor Tu is important for growth and translation. Elongation factor Tu has also been shown to act as a chaperone in *E. coli*, consistent with induction of proteins involved in responding to heat or other protein damaging stresses [35]. Recently, elongation factor Tu has been shown to have a unique post-translational modification that has roles in colonization of the respiratory tract [36,37]. The differential expression of Tu due to *mucE* overexpression suggests there may be signaling networks dependent upon *mucE* that we have not yet been identified. Although, previous studies have shown that the growth rate is slower in mucoid strains and the virulence is increased after deleting AlgU [15,38], the relationship between MucE and growth or virulence need further study. Together, iTRAQ analysis suggests that MucE signaling affected both AlgU-dependent and AlgU-independent protein expression.

## Conclusions

The alternative sigma factor AlgU was responsible for *mucE* transcription. Together, our results suggest there is a positive feedback regulation of MucE by AlgU in *P. aeruginosa,* and the expression of *mucE* can be induced by exposure to certain cell wall stress agents, suggesting that *mucE* may be part of the signal transduction that senses the cell wall stress to *P. aeruginosa*.

## Authors’ contributions

YY designed, performed the experiments, and drafted the manuscript; FHD, TRW and CLP performed the experiments and revised the manuscript; XW and MJS revised the manuscript; HDY designed the experiments and revised the manuscript. All authors read and approved the final manuscript.

## Supplementary Material

Additional file 1 Supplementary materials and methods. Click here for file
