# Supplementary material for: Expression of mucoid induction factor MucE is dependent upon the alternate sigma factor AlgU in Pseudomonas aeruginosa
Source: BMC Microbiol. 2013 Oct 18;13:232. doi: 10.1186/1471-2180-13-232 (PMC3819740; doi:10.1186/1471-2180-13-232)
Supplement: Additional file 1 — Supplementary materials and methods. [file 1471-2180-13-232-S1.doc]

**Supplementary Materials and Methods**

**HA-AlgU Purification and Western Blotting.** *E. coli* TOP10 cells carrying pHERD20T-*algU* were cultured in 6 ml of LB broth supplemented with carbenicillin at a concentration of 100 μg/ml. When OD_600_ was 0.6, L-arabinose was added to a concentration of 0.1%. After 2 hour induction, cells were collected for cell lysis. Following sonication, the protein concentration of the supernatant was measured using the Bio-Rad Dc protein assay reagents (Bio-Rad). HA-AlgU was purified using Pierce HA Tag IP/Co- IP Kit (Thermo scientific) according to the supplier’s instruction. For Western blot detection, protein was mixed with 2×sample loading buffer and separated on a pre-cast SDS-PAGE gel (10-20%, Bio-Rad). After electrophoresis, proteins were transferred to PVDF membrane (GE) for immuno-detection. A primary monoclonal antibody of mouse anti-AlgU was used at a dilution of 1: 1,000, while a goat anti-mouse immunoglobulin G (heavy and light chains) conjugated with horseradish peroxidase (Pierce) (1:5,000) was used as the secondary antibody. The immuno-reactive protein was visualized using the Amersham ECL kit (GE), and the image was photographed in UVP Bioimaging system (UVP, LLC).

**Electrophoretic Mobility Shift Assay (EMSA).** EMSA was performed according to Jones *et al*. with minor modification . Briefly, DNA fragment containing the *mucE* promoter region was generated by PCR amplification and gel extraction (Qiagen). DNA binding reaction was performed in a total of 10 µl volume: 3 µl purified protein combined with 50 ng DNA fragment in EMSA binding buffer (20mM Tris-HCl pH8.0, 200mM 140 NaCl, 20mM MgCl2, 20% glycerol) and 200 ng Poly(deoxyinosinic-deoxycytidylic) acid sodium salt (Sigma). After treating for 20 minutes at room temperature, nucleic acid sample loading buffer 5× (Bio-Rad) was added. Samples were resolved on a 5% Ready Gel TBE (Bio-Rad). Electrophoresis was performed at 100 V for 2 hours in 0.5×TBE electrophoresis buffer. Gel was then stained in ethidium bromide (EB, SIGMA) for 20 minutes. Image was photographed in UVP Bioimaging system (UVP, LLC).

**Supplementary Table S1.** Bacterial strains and plasmids used in this study

| **Strain and plasmid** | **Phenotype and genotype** | **Source or reference** | |
| --- | --- | --- | --- |
| ***P. aeruginosa* strains** |  |  | |
| CF149 | Non-mucoid, *mucA* mutant (125 aa + 3 aa), *algU* suppressor mutant (A61V) | G. Pier^a^ | |
| CF149(+*algU*) | Mucoid, overexpression of *algU*, Gm^R^, CF149 |  | |
| CF1003 | Mucoid, *algU^+^ mucA^-^*, isolated from the lung of a CF mouse |  | |
| CF3715 | Non-mucoid, *algU^+^mucA^+^*, isolated from early lung colonization in CF | D. Speert^b^ | |
| CF4009 | Non-mucoid, *algU^+^mucA^+^*, isolated from early lung colonization in CF | D. Speert^b^ | |
| CF2 | Non-mucoid, *mucA* mutant, *algU* wild type, derived from clinical mucoid strain | | This study |
| CF11 | Non-mucoid, *mucA* mutant, *algU* wild type, derived from clinical mucoid strain | | This study |
| CF14 | Non-mucoid, *mucA* mutant, *algU* mutant, derived from clinical mucoid strain | | This study |
| CF17 | Non-mucoid, *mucA* mutant, *algU* wild type, derived from clinical mucoid strain | |  |
| CF28 | Non-mucoid, *mucA* mutant, *algU* mutant, derived from clinical mucoid strain | |  |
| CF4349 | Non-mucoid, *mucA* mutant, *algU* wild type, derived from clinical mucoid strain | |  |
| FRD1 | Mucoid, *algT^+^mucA22,* clinical strain |  | |
| FRD2 | Non-mucoid, *algT* suppressor mutation, FRD1 |  | |
| PAO1 | Non-mucoid, prototroph | P. Phibbs^c^ | |
| PAO1△*algU* | PAO1*ΔalgU,* an in-frame deletion of *algU* |  | |
| PAO1*mucE*:: ISphoA/hah | Non-mucoid, ISphoA/hah insertion site in *mucE* |  | |
| PAO1△*rpoN* | Non-mucoid, PAO1 in-frame deletion of *rpoN* (PA4462) |  | |
| PAO1*rpoS*:: ISlacZ/hah | Non-mucoid, ISlacZ/hah insertion site in *rpoS* |  | |
| PAO1*rpoF*:: ISphoA/hah | Non-mucoid, ISphoA/hah insertion site in *rpOF* |  | |
| PAO1VE1 | Mucoid, *algU*-overexpressing, Gm^R^, PAO1 | This study | |
| PAO1VE2 | Mucoid, *mucE*-overexpressing, Gm^R^, PAO1 |  | |
| PDO300 | Mucoid, *algT^+^mucA22* in PAO1 |  | |
| PAO579 | Mucoid, *algU^+^mucA^+^*, *pilA108*, derived from PAO381, and PAO1 |  | |
| PAO581 | Mucoid, PAO1 *algU^+^ mucA25* |  | |
| **E. coli strains** |  |  | |
| TOP10 | DH5α derivative | Invitrogen | |
| **Plasmids** |  |  | |
| pRK2013 | Km^R^ Tra Mob ColE1 |  | |
| pHERD 20T | pUCP20T P_lac_ replaced by fragment of *araC*-P_BAD_ cassette |  | |
| pLP170 | Promoterless transcriptional fusion vector contain *lacZ* gene, Ap^R^ | L. Passadorf^d^ | |
| miniCTX-P*_mucE_*-lacZ | P*_mucE_* promoter fused with *lacZ* for integration at the CTX phage att site in P. aeruginosa | This study | |
| pLP170-P*_mucE_* | promotor of *mucE* from PAO1 in _P_LP170 EcoRI/HindIII | This study | |
| pHERD 20T-*mucE* | *mucE* (PA4033) from PAO1 in pHERD20T EcoRI/HindIII |  | |
| pHERD 20T-PAO1*algU* | *algU* (PA0762) from PAO1 in pHERD20T EcoRI/HindIII |  | |
| pHERD 20T-CF149*algU* | *algU* (PA0762) from CF149 in pHERD20T EcoRI/HindIII |  | |
| pHERD 20T-FRD2*algU* | *algU* (PA0762) from FRD2 in pHERD20T EcoRI/HindIII | This study | |
| pHERD 20T-*rpoN* | *rpoN* (PA4462) from PAO1 in pHERD20T EcoRI/HindIII |  | |
| pHERD 20T-HA-*algU* | *algU* (PA0762) from PAO1 in pHERD20T EcoRI/HindIII | This study | |
| pHERD 20T-HA-*rpoD*-His | *rpoD* (PA0576) from PAO1 in pHERD20T EcoRI/HindIII |  | |
| pHERD 20T-*rpoS*-HA | *rpoS* (PA3622) from PAO1 in pHERD20T EcoRI/HindIII |  | |
| pHERD 20T-*rpoF*-HA | *rpoF* (PA1455) from PAO1 in pHERD20T EcoRI/HindIII |  | |

^a^, Harvard Medical School, USA; ^b^, University of British Columbia, CA; ^c^, East Carolina University, USA; ^d^, University of Rochester, USA.

**Supplementary Table S2. MucE induces mucoid conversion in nonmucoid CF isolates based on MucA length and AlgU genotype^a^**.

| Strains | MucA length | AlgU genotype | pHERD20T-*mucE*^a^ |
| --- | --- | --- | --- |
| CF27 | WT (194 aa) | WT (193 aa) | Mucoid (30.83±1.88) |
| CF3715 | WT (194 aa) | WT (193 aa) | Mucoid (70.75±3.26) |
| CF4009 | WT (194 aa) | WT (193 aa) | Mucoid (53.18±2.46) |
| CF2 | Thr121Pro (194aa) | WT (193 aa) | Mucoid (36.73±0.25) |
| CF17 | 143 + 3 aa^b^ | WT (193 aa) | Mucoid (30.74±2.03) |
| FRD2 | 143 + 3 aa^b^ | Asp18Gly (193 aa) | Non-mucoid (9.04±0.72) |
| CF14 | 143 + 3 aa^b^ | Gln30Lys (193 aa) | Non-mucoid (9.62±2.08) |
| CF4349 | 125+3 aa^b^ | WT (193 aa) | Mucoid (68.08±2.83) |
| CF149 | 125 + 3 aa^b^ | Ala61Val (193 aa) | Non-mucoid (13.49±1.10) |
| CF11 | 117 aa^c^ | WT (193 aa) | Non-mucoid (10.51±0.53) |
| CF28 | 117 aa^c^ | Tyr29Cys (193 aa) | Non-mucoid (6.85±0.87) |

^a^, pHERD20T-*mucE* was conjugated into these non-mucoid CF isolates, and then incubated on PIA plates containing carbenicillin and 0.1% L-arabinose at 37°C for 24 hours. Mucoid or non-mucoid phenotypes were observed and the quantity of alginate production was measured (µg/ml/OD_600_) and listed in the brackets. ^b^, the frameshift mutation in MucA results in the fusion of a truncated MucA (125 or 143 amino acids of N-terminal MucA) with an additional 3 amino acids with no homology to the amino acid sequence of wild type MucA. ^c^, the *mucA* mutation (349C>T) in CF11 and CF28 causes formation of stop codon TAA, and all 117 aa residues of CF11 and CF28 are identical to the wild type sequence.

**Supplementary Table S3. Identification of the *mucE* regulated proteins using iTRAQ analysis^#^**

| Accession | Protein description | Peptides (95%) | VE2:PAO1 | | VE2△AlgU:PAO1 | | VE2:VE2△AlgU | |
| --- | --- | --- | --- | --- | --- | --- | --- | --- |
|  |  |  | Fold change | p value | Fold change | p value | Fold change | p value |
| PA4739 | conserved hypothetical protein | 14 | 0.209 | 0.000 | 1.207 | 0.049 | 0.175 | 0.000 |
| PA4277 | elongation factor Tu | 28 | 0.788 | 0.000 | 0.775 | 0.002 | 1.008 | 0.929 |
| PA2300 | chitinase | 10 | 0.693 | 0.001 | 0.935 | 0.446 | 0.738 | 0.003 |
| PA5369 | phosphate ABC transporter, periplasmic phosphate-binding protein, PstS | 13 | 0.308 | 0.002 | 1.226 | 0.122 | 0.271 | 0.003 |
| PA1092 | flagellin type B | 14 | 0.365 | 0.002 | 1.270 | 0.209 | 0.338 | 0.009 |
| PA4315 | transcriptional regulator MvaT, P16 subunit | 11 | 0.786 | 0.008 | 0.716 | 0.000 | 1.100 | 0.265 |
| PA1159 | probable cold-shock protein | 5 | 1.337 | 0.022 | 0.747 | 0.000 | 1.790 | 0.001 |
| PA4922 | azurin precursor | 8 | 0.504 | 0.025 | 1.199 | 0.501 | 0.428 | 0.066 |
| PA4385 | GroEL protein | 13 | 1.283 | 0.029 | 0.825 | 0.031 | 1.572 | 0.010 |
| PA3280 | Pyrophosphate-specific outer membrane porin OprO precursor | 13 | 0.709 | 0.037 | 1.183 | 0.119 | 0.600 | 0.006 |
| PA0888 | arginine/ornithine binding protein AotJ | 5 | 0.556 | 0.046 | 0.775 | 0.081 | 0.738 | 0.186 |
| PA3529 | probable peroxidase | 10 | 0.797 | 0.075 | 0.745 | 0.004 | 1.051 | 0.569 |
| PA3162 | 30S ribosomal protein S1 | 5 | 1.362 | 0.111 | 0.829 | 0.050 | 1.566 | 0.055 |
| PA5046 | malic enzyme | 4 | 1.181 | 0.123 | 0.925 | 0.017 | 1.277 | 0.047 |
| PA4251 | 50S ribosomal protein L5 | 4 | 1.611 | 0.124 | 0.876 | 0.029 | 1.834 | 0.068 |
| PA1580 | citrate synthase | 3 | 1.399 | 0.136 | 1.231 | 0.196 | 1.141 | 0.050 |
| PA4761 | DnaK protein | 13 | 0.933 | 0.443 | 0.751 | 0.010 | 1.236 | 0.078 |
| PA5556 | ATP synthase alpha chain | 11 | 1.147 | 0.457 | 0.851 | 0.040 | 1.319 | 0.154 |
| PA0456 | probable cold-shock protein | 4 | 0.958 | 0.704 | 0.827 | 0.017 | 1.155 | 0.195 |
| PA0139 | alkyl hydroperoxide reductase subunit C | 6 | 0.959 | 0.753 | 0.798 | 0.001 | 1.238 | 0.120 |
| PA5060 | polyhydroxyalkanoate synthesis protein PhaF | 6 | 0.937 | 0.848 | 0.685 | 0.353 | 1.394 | 0.022 |
| PA1494 | conserved hypothetical protein | 43 | 0.999 | 0.994 | 0.802 | 0.011 | 1.215 | 0.065 |

^#^, yellow fonts represent the gene expression between these strains is significant different (p<0.05); green fonts represent the expression of these genes were down-regulated (fold change lower than 1); red fonts represent the expression of these genes were up-regulated (fold change higher than 1); Blue fonts represent these genes are MucE-dependent but AlgU-independent.

**Supplementary Figure S1. MucE positively regulates the activity of P*_algU_* and P*_algD_.*** The promoter activity of P*_algU_*-*lacZ* and P*_algD_*-*lacZ* in pLP170 was measured using Miller assay in PAO1 and PAO1VE2.


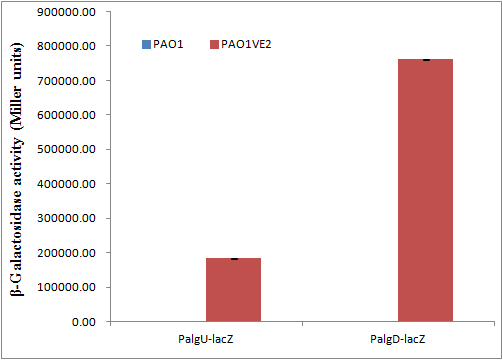


**Supplementary Figure S2. MucE affects AlgU-induced alginate overproduction in *P. aeruginosa* PAO1.** AlgU was induced from pHERD20T-*algU* in PAO1 and PAO1*mucE*:: ISphoA/hah, respectively. MucE was induced from pHERD20T-*mucE* in PAO1 and PAO1Δ*algU*, respectively. Alginate production (µg/ml/OD_600_) was measured after growth for 48 hours at 37 ℃ on PIA plate supplemented with 300 µg/ml of carbenicillin and 0.1% L-Ara.


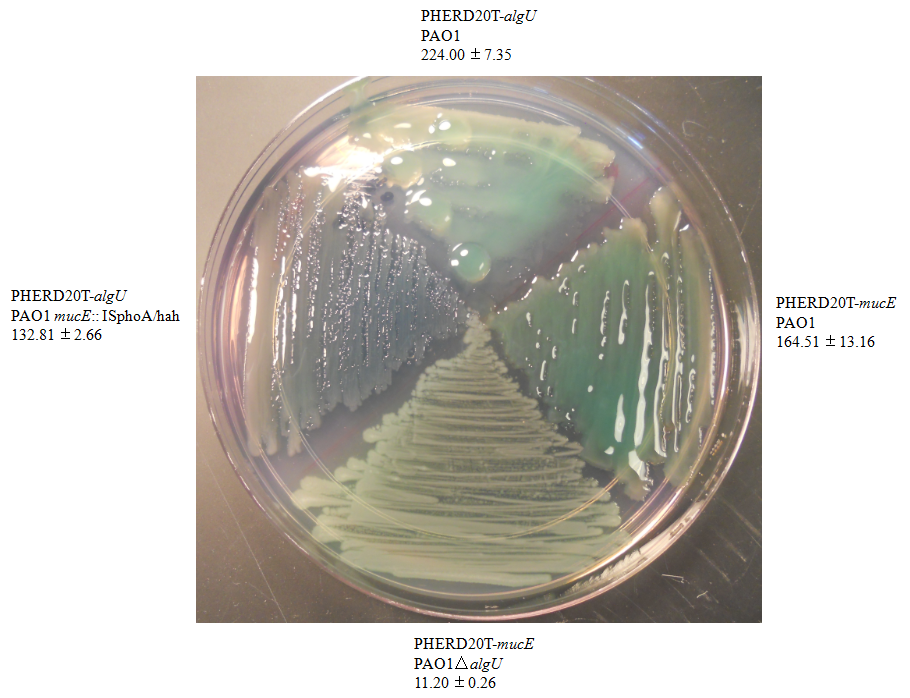


**Supplementary Figure S3. Detection of purified AlgU by Western blot.**

Lane 1: Protein marker; Lane 2: Purified HA-AlgU from *E. coli* TOP10 cells containing pHERD20T-HA-*algU*. Lane 3: Total cell lysate of *E. coli* TOP10 cells carrying pHERD20T-HA-*algU* before purification. Lane 4: Total cell lysates from TOP10 cells containing vector control pHERD20T.

1 2 3 4

**
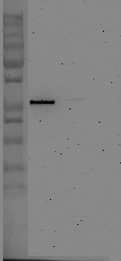
**

50 kD

37 kD

25 kD

AlgU

**Supplementary Figure S4. Purified AlgU binds to the *mucE* promoter region.**

Lane 1: Free DNA of P*_mucE_*; Lane 2: HA-AlgU plus the P*_mucE_* promoter; Lane 3: AlgU plus P*_mucE_* DNA and *E. coli* RNA Polymerase Core Enzyme (Epicentre); Lane 4: HA-AlgU; Lane 5: HA-AlgU plus *E. coli* RNA Polymerase Core Enzyme.

1 2 3 4 5


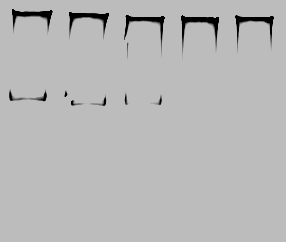


P*_mucE_* DNA

**References:**

1. Schurr MJ, Yu H, Martinez-Salazar JM, Boucher JC, Deretic V: **Control of AlgU, a member of the sigma E-like family of stress sigma factors, by the negative regulators MucA and MucB and *Pseudomonas aeruginosa* conversion to mucoidy in cystic fibrosis**. *J Bacteriol* 1996, **178**(16):4997-5004.

2. Jones CJ, Ryder CR, Mann EE, Wozniak DJ: **AmrZ modulates *Pseudomonas aeruginosa* biofilm architecture by directly repressing transcription of the *psl* operon**. *J Bacteriol* 2013, **195**(8):1637-1644.

3. Yin Y, Withers TR, Wang X, Yu HD: **Evidence for sigma factor competition in the regulation of alginate production by *Pseudomonas aeruginosa***. *PLoS One* 2013, **8**(8):e72329.

4. Qiu D, Eisinger VM, Head NE, Pier GB, Yu HD: **ClpXP proteases positively regulate alginate overexpression and mucoid conversion in *Pseudomonas aeruginosa***. *Microbiology* 2008, **154**(Pt 7):2119-2130.

5. Ohman DE, Chakrabarty AM: **Genetic mapping of chromosomal determinants for the production of the exopolysaccharide alginate in a *Pseudomonas aeruginosa* cystic fibrosis isolate**. *Infect Immun* 1981, **33**(1):142-148.

6. Olson JC, Ohman DE: **Efficient production and processing of elastase and LasA by *Pseudomonas aeruginosa* require zinc and calcium ions**. *J Bacteriol* 1992, **174**(12):4140-4147.

7. Damron FH, Davis MR, Jr., Withers TR, Ernst RK, Goldberg JB, Yu G, Yu HD: **Vanadate and triclosan synergistically induce alginate production by *Pseudomonas aeruginosa* strain PAO1**. *Mol Microbiol* 2011, **81**(2):554-570.

8. Jacobs MA, Alwood A, Thaipisuttikul I, Spencer D, Haugen E, Ernst S, Will O, Kaul R, Raymond C, Levy R *et al*: **Comprehensive transposon mutant library of *Pseudomonas aeruginosa***. *Proc Natl Acad Sci U S A* 2003, **100**(24):14339-14344.

9. Damron FH, Qiu D, Yu HD: **The *Pseudomonas aeruginosa* sensor kinase KinB negatively controls alginate production through AlgW-dependent MucA proteolysis**. *J Bacteriol* 2009, **191**(7):2285-2295.

10. Qiu D, Eisinger VM, Rowen DW, Yu HD: **Regulated proteolysis controls mucoid conversion in *Pseudomonas aeruginosa***. *Proc Natl Acad Sci U S A* 2007, **104**(19):8107-8112.

11. Mathee K, Ciofu O, Sternberg C, Lindum PW, Campbell JI, Jensen P, Johnsen AH, Givskov M, Ohman DE, Molin S *et al*: **Mucoid conversion of *Pseudomonas aeruginosa* by hydrogen peroxide: a mechanism for virulence activation in the cystic fibrosis lung**. *Microbiology* 1999, **145 ( Pt 6)**:1349-1357.

12. Govan JR, Fyfe JA: **Mucoid *Pseudomonas aeruginosa* and cystic fibrosis: resistance of the mucoid from to carbenicillin, flucloxacillin and tobramycin and the isolation of mucoid variants in vitro**. *J Antimicrob Chemother* 1978, **4**(3):233-240.

13. Figurski DH, Helinski DR: **Replication of an origin-containing derivative of plasmid RK2 dependent on a plasmid function provided in trans**. *Proc Natl Acad Sci U S A* 1979, **76**(4):1648-1652.
